# Supplementary material for: Gut Dysbiosis Shaped by Cocoa Butter-Based Sucrose-Free HFD Leads to Steatohepatitis, and Insulin Resistance in Mice
Source: Nutrients. 2024 Jun 18;16(12):1929. doi: 10.3390/nu16121929 (PMC11207001; doi:10.3390/nu16121929)
Supplement: Supplementary file 1 [file nutrients-16-01929-s001.zip › nutrients-2989252-Supplementary.pdf]

Supplementary Figures

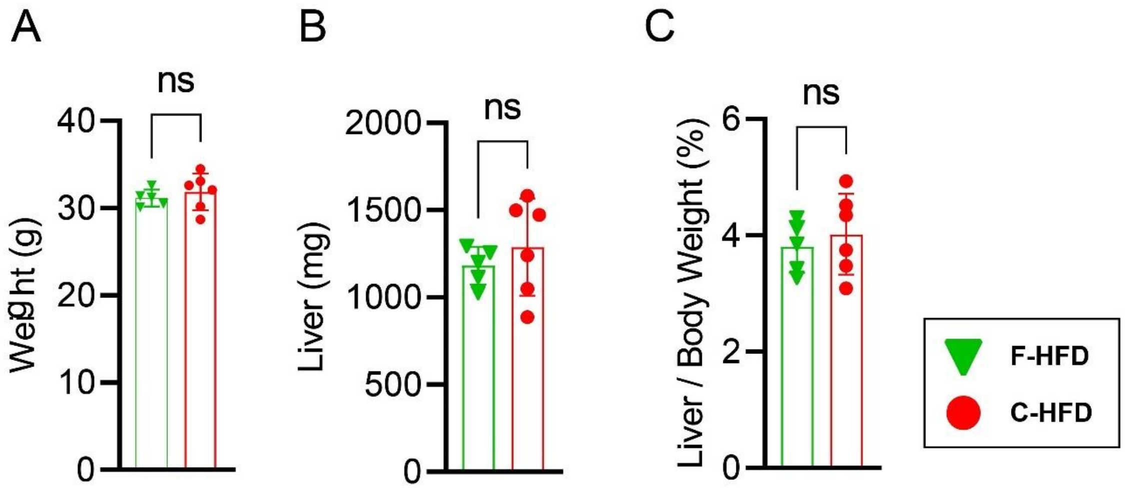

Supplementary Figure 1: (A) After 24 weeks of dietary intervention of sucrose free F-HFD or C-HFD mice body weights were determined. Mice (n=5-6 of each group were scarified after 24 weeks of dietary intervention. (B) Liver weight of the mice were determined. (C)Ratios of liver to body weight (%). Data are shown as means ± SEM. ns= not significant.

Supplementary Table S1: Primer List

Gene Name	Assay ID	Source
Acaca	Mm01304258_m1	Thermo Fisher Scientific
Fasn	Mm00662319_m1	Thermo Fisher Scientific
Scd1	Mm00772290_m1	Thermo Fisher Scientific
Elovl6	Mm00851223_s1	Thermo Fisher Scientific
Pparg	Mm00440940_m1	Thermo Fisher Scientific
Hmgcr	Mm01282499_m1	Thermo Fisher Scientific
Srebp-1	Mm00550338_m1	Thermo Fisher Scientific
CD36	Mm00432403_m1	Thermo Fisher Scientific
Fabp1	Mm00444340_m1	Thermo Fisher Scientific
Ldlr	Mm00440171_m1	Thermo Fisher Scientific
Fatp1	Mm00449511_m1	Thermo Fisher Scientific
Abcg1	Mm00437390_m1	Thermo Fisher Scientific
Mttp	Mm00435015_m1	Thermo Fisher Scientific
Cpt1a	Mm01231183_m1	Thermo Fisher Scientific
Ppara	Mm00440939_m1	Thermo Fisher Scientific
TNF alpha	Mm00443258_m1	Thermo Fisher Scientific
CCL2	Mm00441242_m1	Thermo Fisher Scientific
IL-1b	Mm00434228_m1	Thermo Fisher Scientific
IL-6	Mm00446190_m1	Thermo Fisher Scientific
IL-12b	Mm01288989_m1	Thermo Fisher Scientific
Gapdh	Mm99999915_g1	Thermo Fisher Scientific

Supplementary Table S2: The accession number for each sample

Sample Id	BioSample accession ID	Accession no.	Group	BioProject ID
M16	SAMN37057051	SRR25704418	F-HFD	PRJNA1007291
M17	SAMN37057053	SRR25704417	F-HFD	PRJNA1007292
M18	SAMN37057054	SRR25704416	F-HFD	PRJNA1007293
M19	SAMN37057056	SRR25704415	F-HFD	PRJNA1007294
M20	SAMN37057057	SRR25704414	F-HFD	PRJNA1007295
M21	SAMN37057059	SRR25704421	C-HFD	PRJNA1007296
M22	SAMN37057060	SRR25704412	C-HFD	PRJNA1007297
M23	SAMN37057061	SRR25704411	C-HFD	PRJNA1007298
M24	SAMN37057062	SRR25704410	C-HFD	PRJNA1007299
M25	SAMN37057063	SRR25704409	C-HFD	PRJNA1007300

Supplementary methodology section

Histological Analysis

Immunohistochemistry (IHC) was performed for F4/80 antigen staining. Paraffin-embedded liver tissue sections (4 μm thick) were deparaffinized in xylene and rehydrated using descending grades of ethanol (100%, 95%, and 75%) to water. Antigen retrieval was carried out using target retrieval solution (pH 6.0; Dako, Glostrup, Denmark) by pressure cooker boiling for 8 min and cooling for 15 min. After PBS washing, endogenous peroxidase activity was blocked with 3% H2O2 for 30 min and non-specific antibody binding was blocked with 5% nonfat milk (1 h), followed by 1% bovine serum albumin (BSA) solution (1 h). The samples were incubated overnight at room temperature using primary rabbit polyclonal antibody against F4/80 (1:100 dilution, Abcam® ab100790, pH 6.0, (Cambridge, MA, USA). After washing with PBS (0.5% Tween), samples were incubated for 1 h with secondary, horseradish peroxidase (HRP)-conjugated goat anti-rabbit antibody (EnVision Kit, Dako, Glostrup, Denmark) and color was developed using chromogenic 3,3′-diaminobenzidine (DAB) substrate. Samples were washed in running tap water, lightly counterstained with Harris hematoxylin, dehydrated using ascending grades of ethanol (75%, 95%, and 100%), cleared in xylene, and mounted in dibutylphthalate xylene (DPX). For analysis, digital photomicrographs of adipose tissue sections [20×; PanoramicScan II, 3DHistech, Hungary. URL: https://www.3dhistech.com/products-and-software/hardware/pannoramic-digital-slide-scanners/pannoramic-scan-2/ (accessed on 10 August 2021)] were used to quantify the staining in ten different regions and assess the regional heterogeneity in the tissue samples. The regions were outlined using Aperio ImageScope software [Aperio Vista, CA, USA. URL: https://aperio-imagescope.software.informer.com/9.0/ (accessed on 10 August 2021)].

Aperio-positive pixel count algorithm (version 9) integrated into Imagescope Software was used to quantify the intensity of specific staining in the region. The number of positive pixels was normalized to the number of total pixels (positive and negative) to account for variations in the size of the region sampled. Color and intensity thresholds were set to detect the immuno-staining as positive and the background as negative pixels. Once set, all slides were analyzed using the same parameters. The resultant color markup of the analysis was confirmed for each slide. Liver tissue samples mounted on slides were also processed for Hematoxylin-Eosin (H&E) staining and for Oil Red O staining for fat content following standard protocols as described [1,2].

IHC STAINING PROTOCOL

- Paraffin-embedded sections (4µm thick) of subcutaneous adipose tissue were deparaffinized in xylene and rehydrated through descending grades of ethanol (100, 95, and 75 %) to water. Antigen retrieval was performed by placing slides in target retrieval solution (pH 6.0; Dako, Glostrup, Denmark) in the pressure cooker boiling for 8 min and cooling for 15 min.
- After washing in PBS, endogenous peroxidase activity was blocked with 3 % H_2O_2 for 30 min and non-specific antibody binding was blocked with 5 % nonfat milk for 1 h followed by 1 % bovine serum albumin solution for 1 h.
- The slides were incubated at room temperature overnight with primary antibody (1:800 dilution of rabbit polyclonal anti-F4/80 antibody; Abcam® ab100790). After washing with PBS (0.5 % Tween), slides were incubated for 1 h with secondary antibody (goat anti-rabbit conjugated with horseradish peroxidase (HRP) polymer chain; EnVision™ Kit from Dako, Glostrup, Denmark) and color was developed using 3,3′-diaminobenzidine (DAB) chromogen substrate.
- Specimens were washed in running tap water, lightly counterstained with Harris hematoxylin, dehydrated through ascending grades of ethanol (75, 95, and 100 %), cleared in xylene, and finally mounted in dibutylphthalate xylene (DPX).
- For analysis, digital photomicrographs of four different regions to assess the regional heterogeneity in tissue samples were taken in 20X using PannoramicScan (3DHistech, Hungry). All samples were analysed using imageJ software (NIH,USA).
- Briefly, 10 random fields of the sample are taken in 20X magnification. The jpg images are imported into the Fiji image J application. The images are colour deconvoluted into H-Dab form. Max threshold value is set and applied to all figures. In the Analysis box, area percentage along with mean gray value is selected which will show the size of the IHC image and staining percentage. Once the reading is done for all fields, the analysis file is exported, the readings are averaged and appropriate statistical measures applied. Detailed methodology is given in the reference paper[3].
-

IHC Image J – Quantification Methodology

Briefly, 10 random fields of the sample are taken in 20X magnification. The Jpg images are imported into the Fiji image J application. The images are colour deconvoluted into H-Dab form. Max threshold

value is set and applied to all figures. In the Analysis box, area percentage along with mean gray value is selected which will show the size of the IHC image and staining percentage. Once the reading is done for all fields, the analysis file is exported, the readings are averaged and appropriate statistical measures applied. Detailed methodology is given in the reference paper. Bio-protocol 9(24): e3465. DOI:10.21769/BioProtoc.3465

Oil Red O Quantification

Quantification in ImageJ

- For the quantitative analysis of the ORO staining, convert the RGB image to an 8-bit grayscale image, and then use the image threshold is set using the threshold tool.
- The image is analyzed using the measurement tool. The Area value is the size of the selection within the set threshold. Integrated density gives two results: IntDen, which is the product of Area and Mean gray value, and RawIntDen, which represents the sum of all values of all pixels in the selection. In the Analysis box, area percentage along with mean gray value is selected which will show the size of the ORO image and staining percentage.
- Once the reading is done for all fields, the analysis file is exported, the readings are averaged and appropriate statistical measures applied. Detailed methodology is given in the reference paper[4].

H&E Quantification

Analysis of Inflammation using image J

- For the quantitative analysis of the H&E staining, convert the RGB image to an 16-bit grayscale image, and then use the image threshold is set to the default mode and highlight all the inflammatory cells to be counted and then applied to all to all the slides. The binary image of the particles are analysed according to their circularity. The binary images and side by side compared with their original H&E image for accuracy.

- In the Analysis box, area percentage along with mean gray value is selected which will show the size of the H&E image and staining percentage of the selected cells.
- Once the reading is done for all fields, the analysis file is exported, the readings are averaged and appropriate statistical measures applied.

Analysis of Microvesicular and Macrovesicular using image J

- For the quantitative analysis of the H&E staining, convert the RGB image to an 16-bit grayscale image, and then use the image threshold is set to the intermode parameter and highlight all the vacuoles to be counted and then applied to all to all the slides. The binary image of the particles are analysed according to their circularity. The binary images and side by side compared with their original H&E image for accuracy.
- In the Analysis box, area percentage along with mean gray value is selected which will show the size of the H&E image and staining percentage of the selected cells.
- Once the reading is done for all fields, the analysis file is exported, the readings are averaged and appropriate statistical measures applied[5].

MASSON'S TRICHROME STAINING PROTOCOL

- For direct visualization of collagen fibres and histological assessment of collagen deposition, trichrome staining was performed using the Masson Trichrome Staining Kit (Sigma-Aldrich, St
Louis, MO, USA)

1. Konstantopoulos, P.; Doulamis, I.P.; Tzani, A.; Korou, M.L.; Agapitos, E.; Vlachos, I.S.; Pergialiotis, V.; Verikokos, C.; Mastorakos, G.; Katsilambros, N.L.*, et al.* Metabolic effects of crocus sativus and protective action against non-alcoholic fatty liver disease in diabetic rats. *Biomed Rep* **2017**, *6*, 513-518.
2. Fengler, V.H.I.; Macheiner, T.; Kessler, S.M.; Czepukojc, B.; Gemperlein, K.; Müller, R.; Kiemer, A.K.; Magnes, C.; Haybaeck, J.; Lackner, C.*, et al.* Susceptibility of different mouse wild type strains to develop diet-induced nafld/afld-associated liver disease. *PLOS ONE* **2016**, *11*, e0155163.
3. Crowe, A.R.; Yue, W. Semi-quantitative determination of protein expression using immunohistochemistry staining and analysis: An integrated protocol. *Bio-protocol* **2019**, *9*.
4. Mehlem, A.; Hagberg, C.E.; Muhl, L.; Eriksson, U.; Falkevall, A. Imaging of neutral lipids by oil red o for analyzing the metabolic status in health and disease. *Nature protocols* **2013**, *8*, 1149-1154.
5. Yen, K.; Le, T.T.; Bansal, A.; Narasimhan, S.D.; Cheng, J.X.; Tissenbaum, H.A. A comparative study of fat storage quantitation in nematode caenorhabditis elegans using label and label-free methods. *PLoS One* **2010**, *5*.
